# Genetic signatures of heroin addiction

**DOI:** 10.1097/MD.0000000000004473

**Published:** 2016-08-07

**Authors:** Shaw-Ji Chen, Ding-Lieh Liao, Tsu-Wang Shen, Hsin-Chou Yang, Kuang-Chi Chen, Chia-Hsiang Chen

**Affiliations:** a Institute of Medical Sciences, Tzu Chi University, Hualien; b Department of Psychiatry, Mackay Memorial Hospital, Taitung Branch; c Department of Health Executive Yuan, Bali Psychiatric Center; d Institute of Statistical Science, Academia Sinica, Taipei; e Department of Psychiatry, Chang Gung Memorial Hospital at Linkou; f Department and Graduate Institute of Biomedical Sciences, Chang Gung University, Taoyuan, Taiwan.

**Keywords:** biomarker, diagnosis, genetic signatures, heroin addiction

## Abstract

Heroin addiction is a complex psychiatric disorder with a chronic course and a high relapse rate, which results from the interaction between genetic and environmental factors. Heroin addiction has a substantial heritability in its etiology; hence, identification of individuals with a high genetic propensity to heroin addiction may help prevent the occurrence and relapse of heroin addiction and its complications. The study aimed to identify a small set of genetic signatures that may reliably predict the individuals with a high genetic propensity to heroin addiction. We first measured the transcript level of 13 genes (RASA1, PRKCB, PDK1, JUN, CEBPG, CD74, CEBPB, AUTS2, ENO2, IMPDH2, HAT1, MBD1, and RGS3) in lymphoblastoid cell lines in a sample of 124 male heroin addicts and 124 male control subjects using real-time quantitative PCR. Seven genes (PRKCB, PDK1, JUN, CEBPG, CEBPB, ENO2, and HAT1) showed significant differential expression between the 2 groups. Further analysis using 3 statistical methods including logistic regression analysis, support vector machine learning analysis, and a computer software BIASLESS revealed that a set of 4 genes (JUN, CEBPB, PRKCB, ENO2, or CEBPG) could predict the diagnosis of heroin addiction with the accuracy rate around 85% in our dataset. Our findings support the idea that it is possible to identify genetic signatures of heroin addiction using a small set of expressed genes. However, the study can only be considered as a proof-of-concept study. As the establishment of lymphoblastoid cell line is a laborious and lengthy process, it would be more practical in clinical settings to identify genetic signatures for heroin addiction directly from peripheral blood cells in the future study.

## 1 Introduction

Heroin was synthesized as a legal drug in 1895. It was found to be a highly addictive drug in the early twentieth century, and became popularly abused after the mid-twentieth century.^[1,2]^ Heroin users develop tolerance to heroin quickly and suffer from severe withdrawal symptoms when they stop using heroin. Hence, heroin addicts have a very high relapse rate.^[3,4]^ Heroin addiction became a serious problem in southeastern and southwestern Asia recently.^[5]^ Like that in other countries, heroin users not only had a high recidivism rate but also had the highest mortality rate among the addictive substance users in Taiwan.^[6,7]^

Heroin addiction is a complex disorder resulting from the interactions between genetic susceptibility and environmental factors.^[8]^ Previous studies have demonstrated that the estimated heritability of drug addiction ranges from 0.39 to 0.72 in different drugs.^[9,10]^ In a twin resemblance study for illicit psychoactive substance use, heavy use, abuse, and dependence in a US population, the heritability of illicit psychoactive substance use ranges from 60% to 80%. These findings suggest that genetic factor plays an important role in the pathogenesis of heroin addiction.

Many genes associated with heroin addiction have been reported using different approaches, supporting the high genetic heterogeneity of heroin addiction.^[2,9]^ However, there is still a gap of applying the genetic knowledge of heroin addiction to help manage the patients with heroin addiction in clinical settings. The study aimed to find a small set of genes that can reliably predict the individuals with a high propensity to heroin addiction. The tests of these genes shall be conveniently implemented in the clinical laboratory, and hopefully, the assay of these genes can be used in combination with other social, familial programs to help prevent the occurrence and the relapse of heroin addiction in clinical settings.

## 2 Materials and methods

### 2.1 Subjects

Male adult patients (≥20 years old) met the diagnostic criteria of heroin use disorder according to the Diagnostic and Statistical Manual of Mental Disorders, 5th edition (DSM-5) was recruited into this study. The diagnosis was made based on medical records and interview by senior psychiatrists with consensus. Patients co-morbid with other major psychiatric diagnoses of DSM-5 such as neurodevelopmental disorders, schizophrenia spectrum and other psychotic disorders, bipolar related disorders, depressive disorders, and neurocognitive disorders were excluded. Male adult subjects (≥20 years old) who received regular medical checkups at a local medical center were recruited into this study as controls. A senior psychiatrist evaluated their mental statuses and histories of mental illness. Those who had a history or currently had a diagnosis of major psychiatric disorders according to DSM-5 such as substance-related and addictive disorders, neurodevelopmental disorders, schizophrenia spectrum and other psychotic disorders, bipolar-related disorders, depressive disorders, and neurocognitive disorders were excluded. A total of 8 mL venous blood was collected from each subject for the establishment of lymphoblastoid cell lines (LCLs). Total RNA was extracted from the LCL for the measurement of cDNA of selected genes. The study protocol was approved by the Ethical Committee of Bali Psychiatric Center (approval number: IRB970609-03), and written informed consent was obtained after full explanation of the protocol.

### 2.2 LCL, total RNA, and cDNA preparation

LCL from each subject was established by transforming the lymphocytes with Epstein–Barr virus following the procedures described in our previous paper.^[11]^ After the establishment of LCL, total RNA from the LCL was extracted using TRIZOL Total RNA Isolation Reagent according to manufacturer's instruction (Invitrogen Life Technologies, Carlsbad, CA). The cDNA was prepared using Superscript II RNase H^−^ Reverse Transcriptase following the instructions provided by the manufacturer (Invitrogen Life Technologies). The details were also described in our previous report.^[12]^

### 2.3 Measurement of genetic transcript level in LCL

A total of 13 genes (RASA1, PRKCB, PDK1, JUN, CEBPG, CD74, CEBPB, AUTS2, ENO2, IMPDH2, HAT1, MBD1, and RGS3) were measured in this study. The genetic transcript level was measured using real-time quantitative PCR (RT-qPCR) that was performed by SYBR Green method and implemented in StepOnePlus Real-Time PCR System according to the manufacturer's protocol (Applied Biosystems, Forster City, CA). The detailed procedures were described in our previous report.^[12]^ The relative standard curve method was used for the measurement of the gene transcript level according to the procedure described in User Bulletin #2 ABI PRISM 7700 sequence detection system (Applied Biosystems). In this method, serial dilutions of known amount of RNA from a reference sample (pooled from 40 LCLs of male controls) were used to generate the external standard curve. For each unknown sample, the relative amount was calculated using linear regression analysis from their respective standard curves. The transcript level of measured gene in each subject was normalized by his/her 18S rRNA level. The reference 18S rRNA level was measured using predeveloped TaqMan assay reagents 18S rRNA MGB according to the manufacturer's protocol (Applied Biosystems). All experiments were performed in duplicate. The sequences of all the primer sets and optimal annealing temperature used for RT-qPCR are listed in Table 1.

**Table 1:**
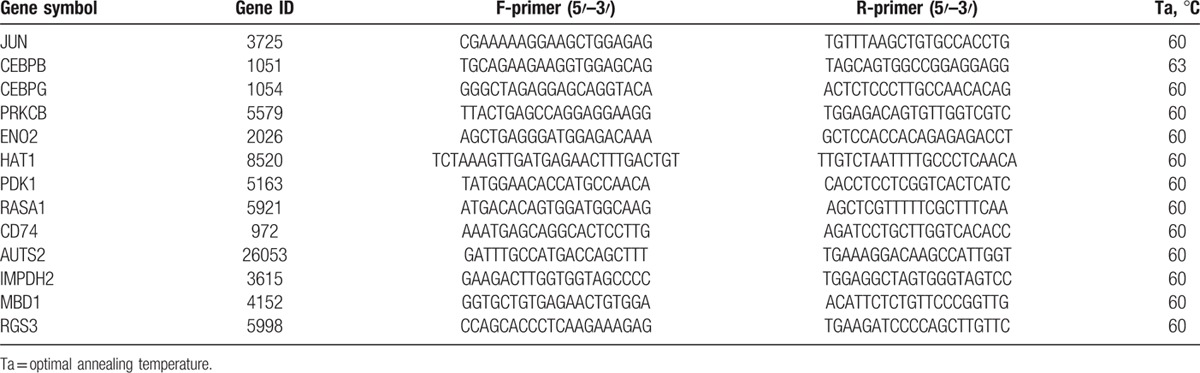
Sequences of primer sets sequences for the real-time quantitative PCR experiments.

### 2.4 Statistical analysis

To cross-validate the findings of genetic signatures for heroin addiction, we used 3 statistical methods to search for the genes that can reliably predict the diagnosis of heroin addiction in this dataset, including multiple logistic regression (LR) analysis, support vector machine (SVM) method, and the online computer program BIASLESS (Biomarkers Identification and Samples Subdivision). LR analysis was implemented using the Statistical Package for the Social Science V18.0 (SPSS Inc., Chicago, IL). The SVM model analysis was implemented using the LIBSVM package by Chang and Lin.^[13]^ The BIASLESS is a software developed for integrative analysis of single nucleotide polymorphisms (SNP) and gene expression, which is an efficient tool for selecting key SNP and gene expression markers and then building models for sample subdivision.^[14]^

## 3 Results

### 3.1 Subjects

All the subjects were Han Chinese from Taiwan. A total of 124 male adults (37.5 ± 9.4 years) fulfilling the diagnostic criteria for heroin use disorders as defined by the DSM-5 were recruited into this study. Also, a total of 124 male adults (42.9 ± 14.6 years) without history or current diagnosis of substance use disorders and other major psychiatric diagnoses were recruited as the control groups. Their LCLs were established, cDNAs were prepared for the genetic assessment.

### 3.2 Genetic assessment

A total of 13 genetic transcript levels were measured for each subject and normalized by the total amount of their 18S rRNA. The mean transcript levels of each of the 13 genes in the patient and control groups are listed in Table 2. Among these 13 genes, 7 genes had a differential expression that reached statistical significance between the 2 groups, including JUN, CEBP, and CEBPG genes that were upregulated and ENO2, PRKCB, HAT1, and PDK1 genes that downregulated in heroin addicts compared with the control group. The mean transcript levels of RASA1, CD47, and IMPDH2 were nominally downregulated in heroin addicts compared with control subjects; however, they did not reach statistic significance after correction for multiple tests. No differences of in the mean transcript levels of AUTS2, MBD1, and RGS3 genes were observed between the 2 groups.

**Table 2:**
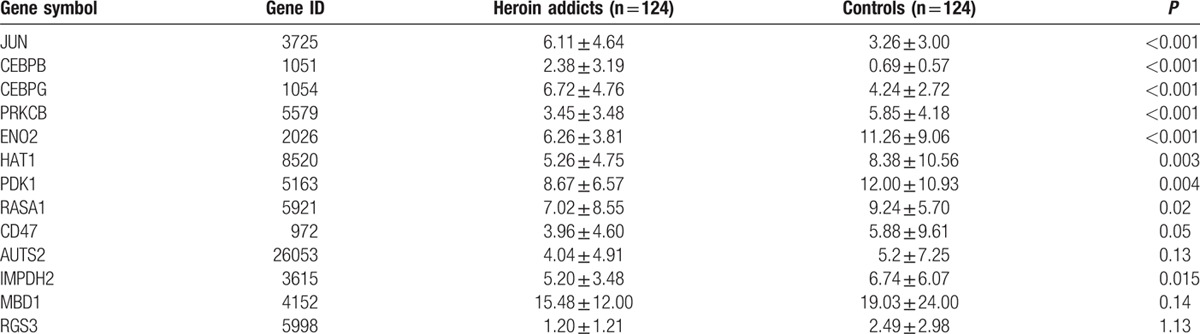
Summary of expression levels of 13 genes in this study.

### 3.3 LR analysis

In LR analysis, subjects with and without the diagnosis of heroin addiction were regressed with a linear combination of the gene expression levels. The accuracy rates of different models containing 13, 7, 5, and 4 genes are listed in Table 3. The odds ratios of the 7 genes that showed significant differential expressions between the 2 groups are listed in Table 4. In this analysis, we found that the 4-gene model that contained JUN, CEBPB, ENO2, and PRKCB genes had an accuracy rate of 85.5% in the prediction of heroin addiction in this dataset.

**Table 3:**
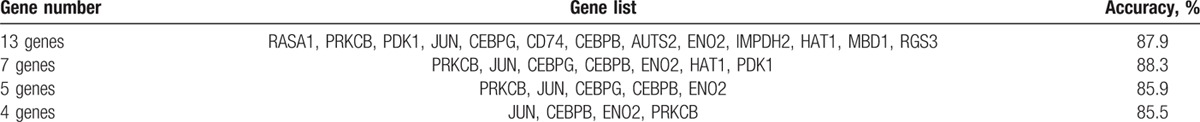
Logistic regression of genetic signature of heroin dependence.

**Table 4:**
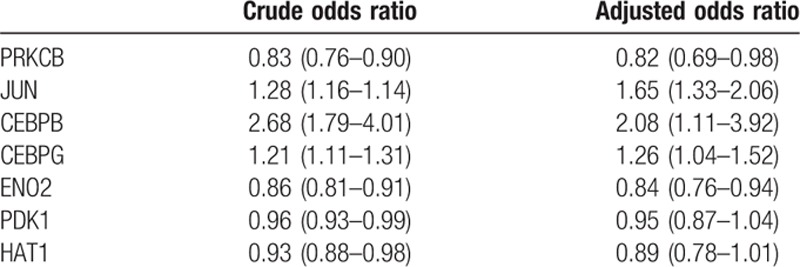
Odds ratio of 7 genes in logistic regression analysis.

### 3.4 SVM analysis

In SVM analysis, we conducted the kernel function as radial basis function as it is the most accepted in a similar analysis. We used leave-one-out cross-validation on the patient gene expression dataset, and all subjects were built-in for both training and testing using the same formula. Individuals in the patient and control groups were randomly partitioned into 10 subsets for a cross-validation. Nine subsets formed a training set and the remaining 1 subset function a testing set. The training set was used to classify individuals in a testing dataset and calculated a testing accuracy. The accuracy rates of different gene combinations are summarized in Table 5. We found that the combination of 4 genes (JUN, ENO2, CEBPG, and PRKCB) had the highest accuracy rate around 84%.

**Table 5:**
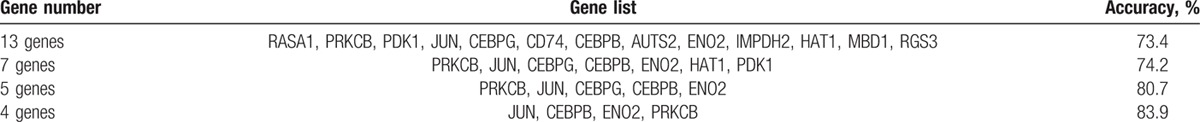
Support vector machines’ analysis of genetic signature of heroin addiction.

### 3.5 BIASLESS analysis

In the analysis using BIALESS software, the data were run with the default parameter values. For each gene, gene expression level was normalized by subtracting the sample mean and dividing by the sample standard deviation of all 248 individuals (from 6 batches). Individuals in the patient and control groups were randomly partitioned into 10 subsets for a cross-validation. Nine subsets formed a training set, and the remaining 1 subset was a testing set. A flexible discrimination analysis was applied to the training set first. The genes with the highest increment of training accuracy were included into the classification model sequentially until the training accuracy reached 1.0 or its increment was less than a threshold of 0.001. Next, the classification model in the training set was used to classify individuals in a testing dataset and calculated a testing accuracy. The previous procedures were repeated until each of the 10 subsets had been analyzed as a testing dataset. Finally, among the 10 candidate classification models, the 1 with the highest cross-validation consistency was selected as the best classification model in this study. A leave-one-out testing accuracy of the best model was calculated.

**Figure 1:**
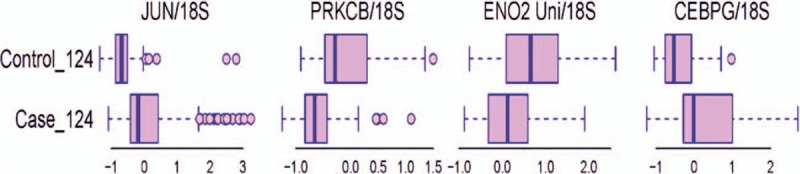
Box-whisker plots of transcript levels of the 4 selected gene expression signatures in the case and control groups.

Our classification analysis identified JUN, CEBPG, ENO2, and PRKCB as the key gene expression signatures for a subdivision of heroin dependence and controls. The suggested classification model with 4 gene signatures had a cross-validation consistency of 4/10 in our 10-fold cross-validation analysis. The average training accuracy rate was 86.7%, and the leave-one-out testing accuracy was 85.5%. Distributions of gene expression levels in the heroin dependence and control groups are displayed in box-whisker plots (Fig. 1).

## 4 Discussion

Heroin addiction, like the other substance use disorders, is a complex disorder resulting from the interplays between environment and genetic predisposition.^[8]^ Hence, identification of the predisposing genes of heroin addiction is crucial to understand the neurobiology and the pathogenesis of heroin addiction. Microarray-based gene expression profiling analysis allows simultaneous measurement of multiple genes expression in cells or tissues,^[15]^ which had been used in discovering genetic pathways related to drug addiction. For example, a set of differentially expressed genes involved in the regulation of transcription, chromatin, and dopamine cell phenotype were detected in the postmortem human brains in chronic cocaine abusers compared with control subjects by microarray and quantitative PCR.^[16]^ Also, altered gene expression in pathways related to T-cell receptor, Janus kinase-signal transducer, and activator of transcription (JAK-Stat) signaling were found among in these 3 groups of alcohol drinkers (alcohol dependence, heavy drinkers, and moderate drinkers) using expression array technology.^[17]^ Our group also conducted a comparative gene expression profiling analysis of LCLs in a small sample of male heroin addicts and male control subjects previously. We detected 924 differentially expressed gene transcripts between these 2 groups, including 279 upregulated and 645 downregulated gene transcripts in individuals with heroin dependence compared with control subjects.^[11]^ These findings support the complex polygenic nature of heroin addiction.

Results from microarray-based gene expression studies usually contain dozens of genes, which are difficult to be applied in clinical settings. Hence, several groups have attempted to use a small set of genes as signatures to represent the findings from microarray-based studies. For example, a 21-gene assay was found helpful to assist in adjuvant treatment decision in hormone receptor-positive breast cancer patients.^[18]^ An 8-gene expression signature could help to predict the survival and time to the treatment of chronic lymphocytic leukemia,^[19]^ and a 6-gene signature was found to be able to distinguish 4 subgroups of neuroblastoma.^[20]^ In drug abuse, no such study was reported yet in the literature to our knowledge. Hence, the study aimed to test the hypothesis whether a small set of signature genes can reliably differentiate heroin addicts from control subjects.

The study is an extension of our previous study of comparative gene expression profiling analysis of LCLs in a small sample of 20 male heroin addicts and 20 male control subjects.^[11]^ We originally intended to validate the differentially expressed genes found in expression microarray using RT-qPCR. Then, we came up with the idea that if we could find a small set of genes that could reliably predict the individuals with a high genetic propensity to heroin addiction, which would be useful in preventing the occurrence and relapse of heroin addiction. To test this hypothesis, we randomly selected 13 genes based on our preliminary study and measured the transcript levels of 13 genes in 124 male heroin addicts and 124 male control subjects using RT-qPCR. Among these 13 genes, we found 7 genes were differentially expressed that reached statistical significance after correcting for multiple testing. These 7 genes were further analyzed for building genetic signatures using 3 different statistical methods. We finally obtained 4-gene signatures that can predict the diagnosis of heroin addiction with the accuracy rate of around 85% in our dataset.

These 4 genes include JUN, CEBPB, ENO2, and PRKCB in LR modeling and SVM analysis while in the analysis using BIALESS computer program, the CEBPB gene was replaced by the CEBPG. The discrepancy between these methods can be attributed to the different algorithms of these methods. Nevertheless, the identification of these genes supports the idea that a small set of gene transcripts can function as genetic signatures of heroin addiction.

The interpretation of the biological significance of these signature genes of heroin addiction can be intriguing. JUN is proto-oncogene and a transcription factor that regulates the expression of its target genes. A recent paper reported that increased c-Jun expression in the cerebellum of rats under long-term heroin administration, which was attributed to be one of the mechanisms underlying the heroin-induced cerebellum neuronal damage.^[21]^ However, no other studies reported elevated intrinsic JUN in heroin addicts to our knowledge. Both CEPBP and CEPBG are members of CEPB family that contain a leucine zipper domain to form homodimer and heterodimer with each other. These 2 transcription factors are involved in many physiological and pathological conditions in humans.^[22–25]^ PRKCB is a member of the protein kinase C (PRKC) family that are threonine-specific protein kinases that play an important role in signal transduction, regulation of gene expression, and control of cell division and differentiation. Studies reported that PRCKB played an essential role in dendritic cell differentiation^[26]^ and was involved in the pathogenesis of autism.^[27,28]^ However, the relation between PRKCB and substance use disorders remains to be studied. Enolase is involved in the glycolysis and gluconeogenesis pathways. Human enolase is a homodimeric protein complex consisted of 2 subunits from 3 isoforms of enolase that are encoded by ENO1, ENO3, and ENO2, respectively. Tannu et al reported increased gamma enolase that was encoded by the ENO2 in the nucleus accumbens of cocaine overdose victims compared with control subjects,^[29]^ suggesting the involvement of the ENO2 in the pathophysiology of cocaine dependence. Our previous study also indicated that ENO2 was associated with heroin dependence.^[11]^ These signature genes may not directly link to heroin addiction at first look; they may represent the complex genetic pathways underlying the genetic disposition of heroin addiction. Hence, a combination of the expression of these signature genes can reliably distinct heroin addiction from control subjects with high accuracy rate as shown in this study.

LR is a powerful modeling tool for predicting the outcome; it assumes a linear relationship between predicting variables and outcome.^[30]^ SVM is a supervised machine learning algorithm that is used for both linear and nonlinear classification.^[13]^ Interestingly, both LR and SVM analyses obtain the same 4 genes that had the accuracy rate of approximately 85.5% and 83.9%, respectively, supporting the robustness of the 4-gene signatures of heroin addiction. In the analysis using BIASLESS, the CEBPB was replaced by the CEBPG. Still, the 4-gene model had the accuracy rate of 85.5% in predicting the diagnosis of heroin addiction.

The study has several limitations. Due to limited resources, we were not able to measure more gene transcripts to find better genetic signatures with higher accuracy rate than the current findings in the prediction of heroin addiction. Also, the relatively small sample size can also affect the predicting power of our model in this study. Furthermore, the gene signatures found in this study may not be specific for heroin addiction, as substance use disorders share some common genetic susceptibility.^[31]^ Hence, the specificity of our findings in this study should be tested in another kind of substance use disorders. Also, the present study only measured the gene expression at mRNA level, which may not represent the gene expression at the protein level. It would be interesting to examine the changes of these signature genes at protein level in the postmortem brains of heroin addicts or the brains of rodents under long-term heroin administration in the future study.

Heroin addiction is a complicated result from interactions between genetic susceptibility and environmental factors. To reduce the confounding environment factors, we used LCLs as our experimental materials instead of peripheral blood cells, because the LCL was cultured in the medium for a long period, the influence of environmental factors was controlled to the minimum. Hence, the genes identified in this study can be considered as trait markers than as state markers. Also, the establishment of LCLs is a laborious and lengthy process, which also limits its clinical use. Hence, this study can only be considered as a proof-of-concept study. We suggest that it would be more useful in clinical settings if the future study can identify a small set of signature genes of heroin addiction from peripheral blood cells rather than from LCL.

In conclusion, the present study suggests the feasibility of detecting a small set of genetic signatures from peripheral tissues that can classify the diagnosis of heroin addiction with reasonable accuracy rate. The approach in this study shall facilitate the search for trait genetic markers and biomarkers of heroin addiction, and promote the translational research of heroin addiction in clinical settings.
